# Application of Thin-Layer Chromatography in Combination with Densitometry for the Determination of Diclofenac in Enteric Coated Tablets

**DOI:** 10.3390/ph12040183

**Published:** 2019-12-16

**Authors:** Wioletta Parys, Alina Pyka-Pająk, Małgorzata Dołowy

**Affiliations:** Department of Analytical Chemistry, Faculty of Pharmaceutical Sciences in Sosnowiec, Medical University of Silesia in Katowice, Jagiellońska 4, 41-200 Sosnowiec, Poland; mdolowy@sum.edu.pl

**Keywords:** pharmaceuticals, diclofenac, non-steroidal anti-inflammatory drugs, TLC, densitometry

## Abstract

Diclofenac belongs to the drug class non-steroidal anti-inflammatory drugs widely used in Europe as well as all over the world. Thus, it is important to conduct research on its quality control of available pharmaceutical preparations like for example enteric coated tablets. Among various analytical techniques, thin-layer chromatography (TLC) is ideal for this task due to their short time analysis, ease of operation and low cost. Hence, the aim of this study was to develop the optimal conditions of analysis and quantitative determination of diclofenac sodium in enteric tablets by using TLC in combination with densitometry. Of all chromatographic systems tested, the best is the one which consists of silica gel 60F_254_ and cyclohexane: chloroform:methanol:glacial acetic acid (6:3:0.5:0.5 *v*/*v*) as the mobile phase, which allows the successful separation of examined diclofenac sodium as active component and the largest number (twelve) of its degradation products as potential impurities of its pharmaceutical products. This indicates that the newly developed method is more effective than previously reported assays by Starek and Krzek. Linearity range was found to be 4.00–18.00 μg/spot for diclofenac sodium. The results of the assay of enteric tablet formulations equals 98.8% of diclofenac sodium in relation to label claim is in a good agreement with pharmaceutical requirements.

## 1. Introduction

Diclofenac is a non-steroidal anti-inflammatory drug (NSAID), which is used in the treatment of small and medium severity pain, chronic inflammatory diseases accompanied by fever and osteoarthritis changes. This drug is commercially available in a few pharmaceutical forms for oral, intramuscular, rectal, intravenous and also external use in order to minimize its adverse effect and to obtain safe and convenient dosing scheme for the patient. However, the most popular pharmaceutical form is a tablet. Some of them are available over the counter (OTC) [1,2,3]. Moreover, the described drug is a member of NSAIDs commonly used in Europe as well as all over the world. Therefore, there is a big necessity to conduct research on its kinetics in the biological system and quality control of available pharmaceutical preparations. Chromatographic techniques, including thin-layer chromatography (TLC) are ideal for this task due to their short time analysis, ease of operation and low cost. TLC is known to have some advantages in comparison with other methods, i.e., ability to analyze several samples simultaneously, and using small amounts of solvents as mobile phase components, reducing analysis time and costs. Different analytical methods for the determination of diclofenac sodium or potassium in drugs are currently known. These are: thin-layer chromatography [4,5,6,7,8,9,10,11,12,13,14,15,16,17,18], high-performance liquid chromatography (HPLC) [19,20], ultra high performance liquid chromatography (UPLC) [21], gas chromatography [22], capillary electrophoresis [23], spectrofluorimetry [24,25], UV-Vis spectrophotometry [26,27], flow extraction [28], flame emission [29], potentiometry [30,31], voltammetry [22,32,33], scanning colorimetry [34] and the photometric method with diffuse reflection [35]. The American, European and Polish Pharmacopoeias recommend a titration method for the quantitative determination of diclofenac sodium in the pharmaceuticals [36,37,38]. While the pharmacopoeial methods for the assessment the impurities of diclofenac are based on HPLC method. Although, the American Pharmacopoeia indicates the need of confirmation the presence one of the impurities, namely diclofenac related compound A (1-(2,6-dichlorophenyl)-1,3-dihydro-2H-indol-2-one) [36]. However, the Polish and European Pharmacopoeias show the four additional impurities of diclofenac sodium such as 2-[(2,6-dichlorophenyl)amino]-benzaldehyde (impurity B), [2-[(2,6-dichlorophenyl)amino]-phenyl]methanol (impurity C), 2-[2-[(2-bromo-6-chlorophenyl)amino]phenyl]acetic acid (impurity D) and 1,3-dihydro-2H-indol-2-on (impurity E) [37,38]. In addition to this, Polish Pharmacopoeia shows the sixth impurity of this drug namely N-(4-chlorophenyl)-2-(2,6-dichlorophenyl) acetamide (impurity F). The literature review indicates that TLC and HPTLC methods have been used for quantitative determination of diclofenac in both, i.e., simple and complex preparations [4,5,7,14]. Diclofenac sodium was analyzed in different pharmaceutical formulations together with lidocaine [6,7], misoprostol [7], salicylic acid and methyl salicylate [14], tolperisone hydrochloride [8], ibuprofen [9], acetaminophenone (paracetamol) [10,12,15], chlorzoxazone and acetaminophenone [16], chlorzoxazone, acetaminophenone and famotidine [17].

Diclofenac sodium was also determined from serum by HPTLC [13]. This method after appropriate modifications (if required) can further be used for the estimation of this drug in other biological fluids like plasma and urine. The use of TLC is also described, but only for the identification of diclofenac sodium next to paracetamol in urine [18].

A very important element in the validation of analytical method is determination of its selectivity. The selectivity is a good measure of a stationary phase ability to discriminate between two components in any chromatographic technique or mode. However, the selectivity does not say all about how good the separation is between two components since it does not account for the zone spread. For this purpose, resolution (Rs) is the ultimate measure, based on expressions accounting both for selectivity and band spread [39,40]. In the case of works, in which diclofenac sodium to be determined among other substances, the selectivity was assessed on the basis of separation of diclofenac from other substances to be determined. In accordance with International Council for Harmonization of Technical Requirements for Pharmaceuticals for Human Use (ICH) the selectivity of method is based on the development of chromatographic conditions enabling to separate the determined substances from their potential impurities [41]. In the second variant, the standard of examined substance may be subjected to conditions for its stress degradation and then the degradation (decomposition) products may be separated from standard. Of all cited papers, only Starek and Krzek examined the selectivity of the method for the determination of diclofenac sodium in various simple and combined pharmaceutical preparations [7]. In these studies they used the rarely applied aluminum TLC plates precoated with silica gel 60 WF_254_ and mobile phase consisted of cyclohexane: chloroform:methanol in volume composition 12:6:1. The selectivity was based on the separation of reference standard of diclofenac sodium from its impurities A and E, for which R_F_ values were: 0.32, 0.67 and 0.21, respectively. What is more, they evaluated the effect of pH and temperature on the amount of diclofenac in extracts of proper drugs (i.e., tablets, ampoules and capsules). They found a decrease in the content of diclofenac sodium in solution with added HCl because its degradation product, i.e., impurity A appeared. The extracts of proper drugs were treated with UV light at 254 nm and 366 nm. Under these conditions, they found two unidentified degradation products of diclofenac sodium at R_F_ values of 0.19 and 0.23.

The aim of this work was to develop optimal conditions of analysis and quantitative determination of diclofenac sodium in enteric coated tablets by thin-layer chromatography in combination with densitometry as well as to validate this method with particular emphasis on selectivity. Therefore, in our work we searched for such conditions of TLC analysis that would allow us to separate as much as possible the degradation products of diclofenac sodium including related compound A recommended by American Pharmacopoeia [36]. Moreover, by using the spectrodensitometric analysis, the identity of diclofenac was also examined.

## 2. Results and Discussion

### 2.1. Validation of TLC Method

Summarized results of the validation method are presented in Figures S1–S6 as well as in Tables S1–S3, and consequently in next subsections.

#### 2.1.1. Optimization and Selectivity

The separation of diclofenac sodium from degradation substances was carried out by choosing the optimal conditions for TLC analysis. To select the one that will enable the best separation of the various substances mentioned above, i.e., eleven mobile phases (I–XI) given in the Materials and Methods part were tested.

Stability of diclofenac sodium was investigated under stress conditions, i.e., acidic and alkaline hydrolysis, as well as exposure to oxidizing agent and UV radiation, i.e., photodegradation. These solutions of diclofenac sodium were prepared in order to select the most preferred mobile phases as described in Materials and Methods. Solutions of diclofenac sodium in acidic and alkaline medium, as well as with addition of hydrogen peroxide after heating at 90 °C for 2 h, and also methanolic solution of diclofenac sodium after exposure to UV radiation (λ = 254 nm) for 2 h were analyzed using the individual mobile phase.

In addition, diclofenac sodium was exposed to UV radiation (λ = 254 nm) for 1–5 h directly on silica gel. Based on the conducted investigations it was stated that the XI mobile phase, i.e., cyclohexane:chloroform:methanol:glacial acetic acid (6:3:0.5:0.5 *v*/*v*) proved to be the best for investigations of chemical stability of diclofenac sodium. It can be observed that the decomposition products of diclofenac sodium were located in this phase below the front of the mobile phase. This phase also allows for a good separation of the decomposition products. Moreover, suitability of mobile phase XI confirmed the results of additional parameter, i.e., peak resolution, namely *R_s(b)_* calculated by using Equation. 1 for analyzed diclofenac and its related compounds (potential impurities), which have been produced under various stress conditions. The data of R_S_ values listed in Tables S1–S4 (see Supplementary Materials) indicated the in almost cases the R_S_ was satisfactory (>1).

While in the remaining phases, the decomposition product of diclofenac sodium solution exposed to UV radiation did not separate well from the band of diclofenac sodium.

The largest number (from two to six) of separated degradation products of diclofenac sodium was found using this mobile phase, depending on the stress conditions to which diclofenac sodium was subjected. The R_F_ of diclofenac sodium using the XI mobile phase was 0.45 (±0.02).

**Figure 1:**
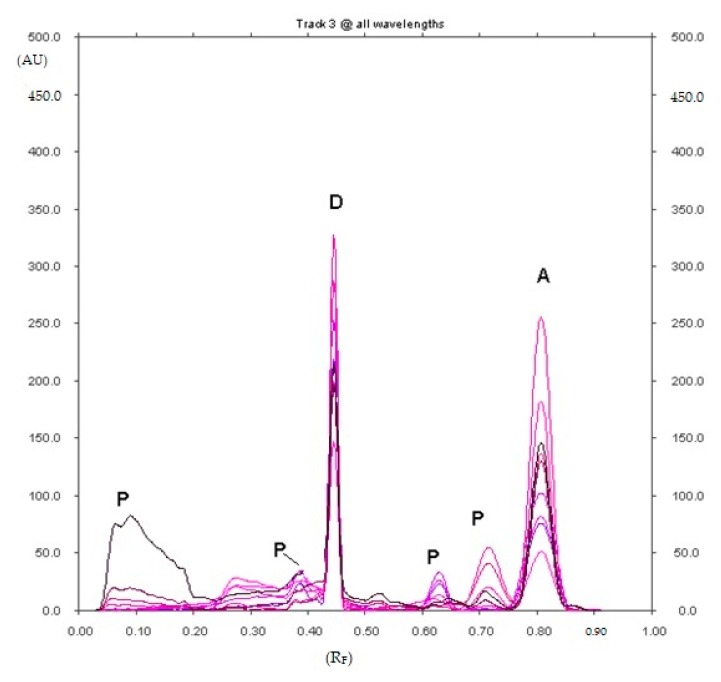
Densitogram of diclofenac sodium with the addition of hydrochloric acid, which was heated at 90 °C for 90 min and developed in the XI mobile phase (D—diclofenac sodium, A—diclofenac related compound A (1-(2,6-dichlorophenyl)-1,3-dihydro-2H-indol-2-one) and P—unidentified degradation products of diclofenac sodium with R_F_ equal to about 0.11, 0.38, 0.63 and 0.73).

**Figure 2:**
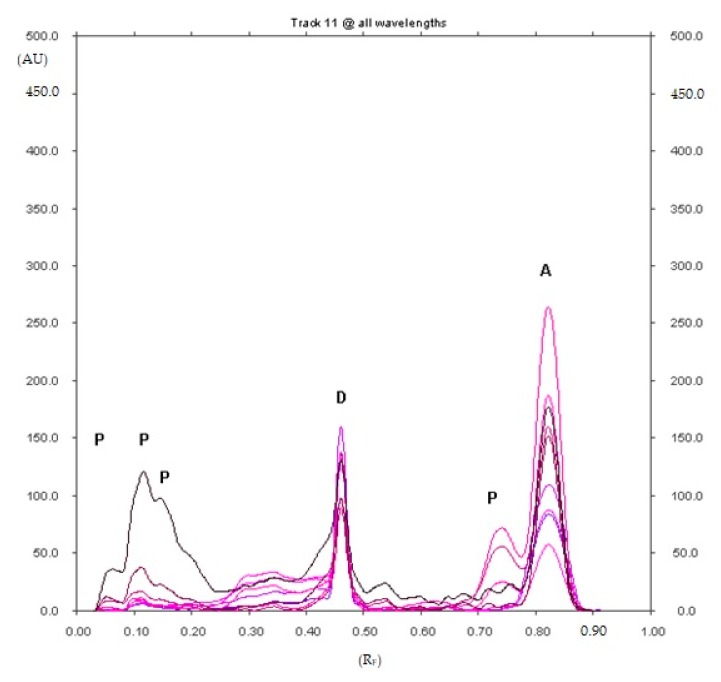
Densitogram of diclofenac sodium with the addition of hydrochloric acid, which was heated at 90 °C for 5 h and developed in the XI mobile phase (D—diclofenac sodium, A—diclofenac related compound A (1-(2,6-dichlorophenyl)-1,3-dihydro-2H-indol-2-one) and P—unidentified degradation products of diclofenac sodium with R_F_ equal to about 0.07, 0.11, 0.16 and 0.73).

Five decomposition products of diclofenac sodium with R_F_ equal to about 0.11, 0.38 0.63, 0.73 and 0.80 were found after 90 min of heating at 90 °C (Figure 1), and the presence of five decomposition products of diclofenac sodium with R_F_ equal to about 0.07, 0.11, 0.16, 0.73 and 0.80 were also found after 5h of heating at 90 °C (Figure 2) on the densitogram of diclofenac sodium with the addition of hydrochloric acid. The decomposition product with R_F_ = 0.80 (±0.02) was identified as diclofenac related compound A (1-(2,6-dichlorophenyl)-1,3-dihydro-2H-indol-2-one). It is known, that diclofenac sodium degrades to the diclofenac related compound A in an acidic medium [7,36]. It was observed, that the content of diclofenac sodium decreased, while the content of the degradation product, i.e., diclofenac related compound A increased as the heating time of diclofenac sodium increases in an acidic medium. The product with R_F_ = 0.63 simultaneously disappeared. The most important results regarding the selection of the mobile phase are presented in the Supplementary Materials.

**Figure 3:**
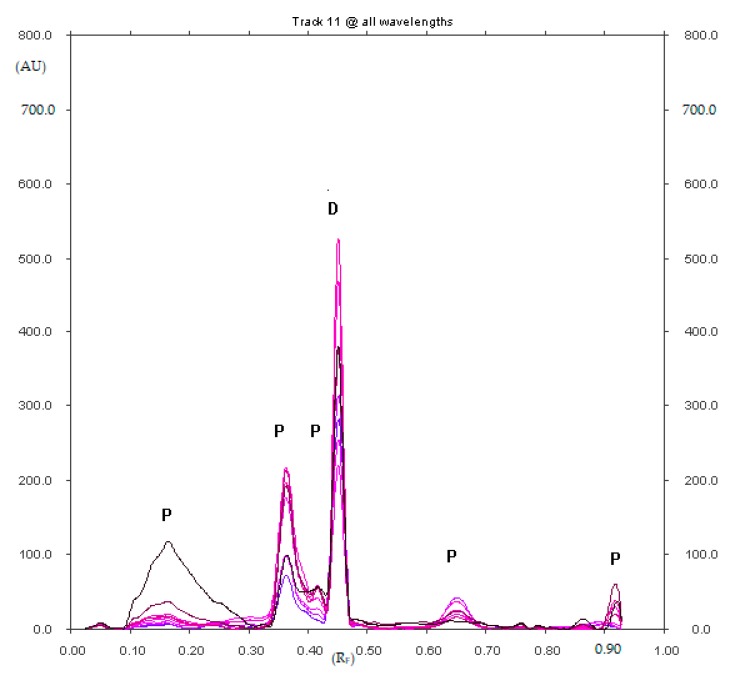
Densitogram of diclofenac sodium from its methanolic solution, which was exposed to UV radiation (λ = 254 nm) for 5 h and developed in the XI mobile phase (D—diclofenac sodium and P—unidentified degradation products of diclofenac sodium with R_F_ equal to about 0.18, 0.37, 0.42, 0.65 and 0.91).

However, five decomposition products of diclofenac sodium from its methanolic solution, which was exposed to UV radiation (λ = 254 nm) for 5 h with R_F_ equal to about 0.18, 0.37, 0.42, 0.65 and 0.91 were observed (Figure 3). The decomposition product with the largest area had R_F_ = 0.37 (±0.02).

**Figure 4:**
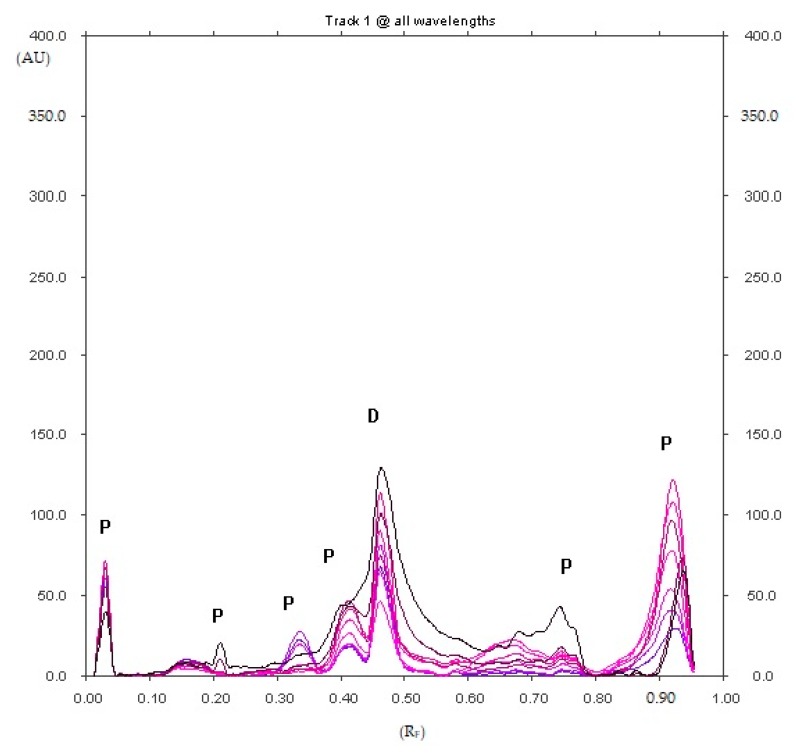
Densitogram of diclofenac sodium, which was exposed to UV radiation (λ = 254 nm) on silica gel for 5 h and developed in the XI mobile phase (D—diclofenac sodium and P—unidentified diclofenac sodium degradation products with R_F_ equal to about 0.03, 0.21, 0.33, 0.41, 0.75 and 0.91).

Confirmation of the right choice as the XI optimal mobile phase is the fact that only this mobile phase allows us to separate the largest number of degradation products of diclofenac sodium directly exposed to UV radiation (λ = 254 nm) on silica gel (Figure 4). Six spots of the substances being degradation products of diclofenac sodium with R_F_ equal to about 0.03, 0.21, 0.33, 0.41, 0.75 and 0.91 were observed. 

Summing up, the stability studies of diclofenac sodium showed that this compound in methanolic solution degrades under an acidic hydrolysis with addition of hydrochloric acid and UV radiation.

Decomposition of diclofenac sodium under alkaline hydrolysis with addition of sodium hydroxide and under oxidation with hydrogen peroxide has not been confirmed. These results are confirmation of previously conducted researches by Azougagha et al. [42] and Elzayat et al. [21]. Conducted studies have shown the instability of the solution of diclofenac sodium under the influence of UV radiation. Ioele et al. [43] studied the stability of diclofenac sodium on UV radiation in a pharmaceutical formulation as a gel. Studies of these scientists have also shown the instability of this compound on UV radiation. A greater degradation of diclofenac sodium in an acidic medium in relation to the exposure on UV radiation (λ = 254 nm) of solution of diclofenac sodium has been observed.

Studies concerning the stability of diclofenac sodium on silica gel have also be conducted. The stability of diclofenac sodium at temperature of 40 °C and UV radiation at 254 nm was studied. No decomposition products were detected in the sample exposed to elevated temperature, therefore diclofenac sodium was stable at 40 °C. Our studies have shown the instability of diclofenac sodium exposed to UV radiation (λ = 254 nm) on silica gel. Spots of diclofenac sodium on silica gel and exposed to UV radiation (λ = 254 nm) clearly yellowed. It can see the decomposition products after just one hour of exposed to UV radiation (λ = 254 nm). The percentage of the band area of diclofenac sodium decreases during exposition to UV radiation (λ = 254 nm). Studies on the stability of diclofenac sodium on silica gel have not been previously described in the scientific literature.

We concluded that the XI optimal mobile phase allowed us to detect six decomposition products of diclofenac sodium in an acidic medium (with R_F_ equal to about 0.07, 0.11, 0.38, 0.63, 0.73 and 0.80). A maximum of five decomposition products of diclofenac sodium (with R_F_ equal to about 0.18, 0.37, 0.42, 0.65 and 0.91) were found in the methanolic solution of diclofenac sodium, which was exposed to UV radiation (λ = 254 nm). While the presence of six degradation products of diclofenac sodium (with R_F_ equal to about 0.03, 0.21, 0.33, 0.41, 0.75 and 0.91) was found in the case of its exposure to UV radiation (λ = 254 nm) directly on silica gel. It should be stated that the XI mobile phase allows to detect a total of twelve decomposition products of diclofenac sodium by analyzing their the R_F_ values under various conditions (acidic medium and UV radiation). While the methodology for detection of impurities indicated by Polish and European Farmakopeas using HPLC technique allows to detect six and five impurities of diclofenac sodium, respectively [37,38].

**Figure 5:**
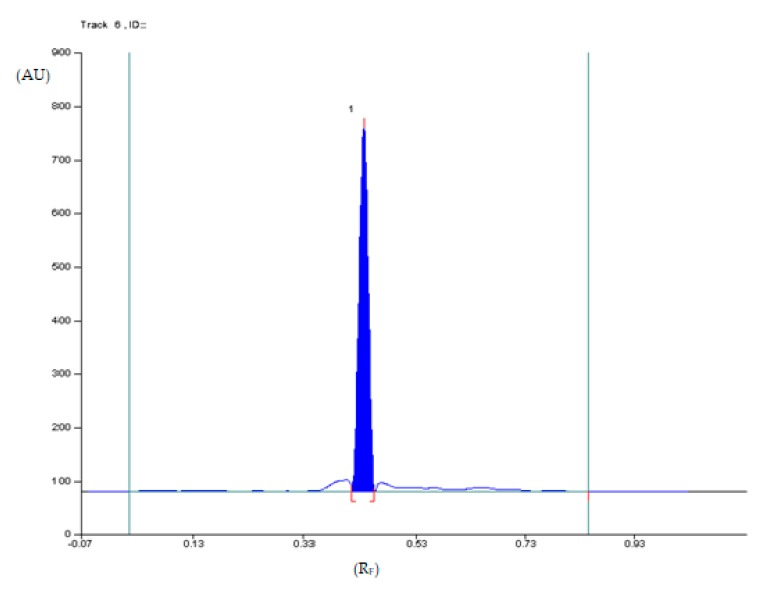
Densitogram of diclofenac sodium extracted from enteric tablets obtained by using thin-layer chromatography (TLC) analysis and the XI mobile phase.

**Figure 6:**
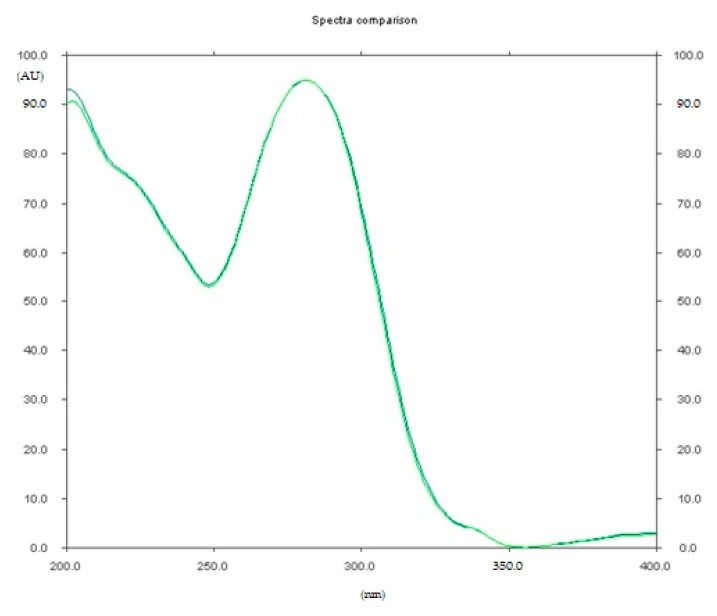
Comparison of the spectrodensitograms of diclofenac sodium standard at concentration of 12 mg/5 mL and diclofenac sodium extracted from enteric tablets obtained by using TLC analysis and the XI mobile phase.

The densitogram of diclofenac sodium (Figure 5) indicates that the TLC technique combined with densitometry used to quantify diclofenac sodium in Diclac 50 enteric tablets (produced by Sandoz) is highly selective. The mean value of R_F_ of diclofenac sodium was 0.45 (±0.02) and it was consistent with the R_F_ value, which was achieved for this active substance coming from tablets extract. Similarly, the spectrodensitograms of diclofenac sodium standards are similar to those derived from studied tablets sample (Figure 6).

No diclofenac sodium degradation products as well as other impurities were found in the pharmaceutical preparation Diclac 50. 

#### 2.1.2. Accuracy

The accuracy of the method was evaluated by measurement of recovery (Table 1). When accurately known amount of diclofenac sodium was added to powdered tablets containing a proper quantity of diclofenac sodium, the recoveries from commercial enteric tablets were placed in the range of 99.4–100.4% (Table 1). The low coefficient of variation values (CV < 3%) was indicative of the accuracy of the method. Moreover, the obtained accuracy was comparable to the mean results of accuracy of HPLC method developed for the determination of diclofenac potassium in oral suspension (RSD% = 98.28–101.95) and better in relation to accuracy of HPLC procedure described for analysis of diclofenac sodium in form of commercial tablets [44]. The mean percentage of recovery of examined active ingredient in tablets was 95.2 ± 4.9% [45].

#### 2.1.3. Calibration and Range

The statistical data shown in Table 1 indicate that fine linear relationship exists between measured peak area (AU) and concentration of diclofenac sodium (μg/spot). The plot was linear in the following range: 4.00–18.00 μg/spot (i.e., 0.8–3.6 μg/μL), thus it is relatively shorter to that presented for HPLC method (10–200 μg/mL) [45]. In addition to this, the graph of residuals against the concentration of diclofenac sodium was also plotted. It can be observed that the residuals were distributed above and below the zero residuals line, thus it confirms the linearity of proposed TLC method (Supplementary Materials).

#### 2.1.4. Precision

The precision (repeatability and intermediate) of the proposed method was studied at three different concentrations of diclofenac sodium obtained from tablet extractions. The results from these experiments, expressed as the coefficients of variation (CV, %) of the respectively response factors (a relationship between the peak area and concentration of diclofenac sodium) are presented in Table 1. Since CV values for repeatability and intermediate were <3% the method was precise. In the case of HPLC method dedicated for the analysis of examined drug substance in oral suspension, the repeatability and intermediate precision expressed as relative standard deviation was 1.21% and 0.85%, respectively [44]. Thus, it was less than 2%. However, a newly developed (in 2019) HPLC method for the determination of diclofenac in tablets is characterized by %CV of interday and intraday precision less than 7% [45].

#### 2.1.5. Limit of Detection (LOD) and Limit of Quantification (LOQ) Based on the Calibration Curve

The limits of detection and quantification were 0.28 μg/spot and 0.84 μg/spot for diclofenac sodium. Low LOD and LOQ values confirmed the sensitivity of the proposed method.

The LOD and LOQ results obtained in this paper showing the TLC-densitometric method sensitivity. The detection limit is much higher in comparison with the HPLC method (12.5 ng/mL) [45], but it is enough for the determination of diclofenac in studied enteric coated tablets.

#### 2.1.6. Robustness

The standard deviation of peak areas measured for each of the five parameters, which were changed (see Section 3.8.6) in the conducted experiment in order to check the robustness of applied method was placed in the range: 0.78–1.35% (Table 2). The low value of % RSD (<2%) indicates the robustness of proposed TLC-densitometric methods during its normal use.

#### 2.1.7. Analysis of Diclofenac Sodium in Commercial Enteric Tablets

In each case, the R_F_ values of diclofenac sodium standard, and those coming from commercial enteric tablets were equal to 0.45 ± 0.02. The identities of diclofenac sodium standard and from samples (commercial enteric tablets) were also confirmed by analysis of their spectra. A very good correspondence between both spectrodensitograms was stated. In all cases the absorption maxima (λ_max_) were equal to 278 nm. The purities of peaks of sodium diclofenac standards, and samples were also assessed by comparing the spectra at the peak start (S), peak apex (M) and peak end (E) of spot. It was found that r(S, M) > 0.999, and r(M,E) > 0.999 for all analyses performed by proposed TLC-densitometric technique. Statistical data showing the results of quantitative determination of diclofenac sodium obtained on the basis of ten repeated different analyses of pharmaceutical preparation are presented in Table 3. It was stated that diclofenac sodium amounts in commercial enteric tablets, which were determined by the TLC-densitometric method, were equal to 98.8% in the relation to label claim. It fulfills the demand the percent content required by American Pharmacopoeia for diclofenac sodium as an active ingredient. According to American Pharmacopeia the content of diclofenac sodium in tablets should be in the range 90–110% [36]. The results obtained by means of proposed TLC-densitometric method were comparable with diclofenac content determined with the use of HPLC procedures in oral suspension and tablets [44,45]. This proved that the proposed method was an alternative for the determination of diclofenac in enteric coated tablets.

## 3. Materials and Methods

### 3.1. Apparatus

Densitometer: Camag (Muttenz, Switzerland) TLC Scanner 3 with winCATS 1.4.2 software. IKA Ultra-Turrax® Tube Drive Workstation with BMT-20-S Tube for grinding with balls of stainless steel. NP-TLC plates: 10 cm × 20 cm glass plates precoated with 0.20 mm layers of silica gel 60F_254_ (E. Merck, Germany, # 1.05554) and TLC silica gel 60F_254_ plates (E. Merck, Darmstadt, Germany, # 1.05570).

The 5 μL Camag micropipettes (Muttenz, Switzerland) were used to apply the solutions onto the plates.

Chromatographic chamber: twin-trough chamber for 20 cm × 10 cm plates (#0.222.5254, Camag, Muttenz, Switzerland).

### 3.2. Pharmaceutical Reference Standards and Chemicals

Diclofenac sodium (European Pharmacopoeia (EP) Reference Standard, Sigma-Aldrich, St. Louis, MO, USA), and diclofenac related compound A (United States Pharmacopeia (USP) Reference Standard) were used as standards. All chemicals and reagents for the TLC method were analytical grade and were purchased from POCh (Gliwice, Poland).

### 3.3. Pharmaceutical Preparation

Pharmaceutical preparation, namely: Diclac50 enteric coated tablets (Sandoz, Kundl, Austria) containing diclofenac sodium (50 mg) in one commercial enteric tablet was used in this study.

### 3.4. Preparation the Sample of Tablets

Ten tablets were ground for 9 min with a speed equal to 9000 rpm using an IKA Ultra-Turrax® Tube Drive Workstation with a BMT-20-S tube for grinding with balls of stainless steel. After this time, the obtained powders of commercial enteric tablets (equivalent to 100 mg diclofenac sodium by weighing the powder to an accuracy of 0.1 mg) were extracted using 10 mL of methanol for 25 min with a speed equal to 6000 rpm using an IKA Ultra-Turrax® Tube Drive Workstation. After extraction, the solutions were filtered through a medium-density filter (Filtrak 389, Bärenstein, Germany) to volumetric flasks (25 mL) and replenished with the use of methanol to demanded volume. By the use of obtained extracts the following working solutions at the concentration of active substance equal to 16.00 mg, 10.00 mg and 6.00 mg in 5 mL for diclofenac sodium were prepared. Of each solution 5 µL was used for the TLC-densitometric analysis and quantitative determination of diclofenac sodium in examined commercial enteric tablets.

### 3.5. Preparation of Standard Solutions

Standard solutions of diclofenac sodium and diclofenac related compound A were prepared by dissolving the solutes in methanol.

### 3.6. Thin Layer Chromatography

Normal phase thin layer chromatography (NP-TLC) was done on TLC silica gel 60F_254_ plates (E. Merck, Germany, # 1.05554). Additionally, TLC silica gel 60F_254_ plates (E. Merck, Germany, # 1.05570) were used for the robustness test. The plates were prewashed with methanol and dried for 24 h at room temperature. Before use, the plates used in NP-TLC were activated at 120 °C for 10 min.

The solutions of pharmaceutical samples and standards of active substances (5 μL) were spotted on the chromatographic plates. The mixture of cyclohexane:chloroform:methanol:glacial acetic acid (6:3:0.5:0.5, *v*/*v*) was used as mobile phase. Of mobile 50 mL was used in all cases. After saturation of twin-trough chamber of 20 cm × 10 cm (#0.222.5254, Camag, Muttenz, Switzerland) with the mobile phase vapor for 30 min., the plates were developed vertically at room temperature (20 °C) to a distance of 7.5 cm and then dried for 20 h at room temperature (20 °C) in a fume cupboard.

### 3.7. Densitometric and Spectrodensitometric Study

Both densitometric and also spectrodensitometric measurements were conducted by a TLC Scanner 3 (Camag, Switzerland) operated in the absorbance mode and controlled by winCATS 1.4.2 software. The radiation source was a deuterium lamp emitting a continuous UV spectrum between 190 and 450 nm. Densitometric scanning was then performed at multi-wavelength in the range of 200–400 nm, at wavelength intervals of 25 nm at each step. Finally, densitometric scanning was then performed at absorption maximum equal to 278 nm for diclofenac sodium. The slit dimensions were 12.00 mm × 0.40 mm, Macro. The optical system was light; the scanning speed was 20 mm/s; the data resolution was 100 μm/step; the measurement type was remission and the measurement mode was absorption.

The chromatographic bands obtained on the densitograms were investigated by spectrodensitometric analysis under the following conditions: the slit dimensions were 12.00 mm × 0.40 mm, macro; the optimal system was resolution; the scanning speed was 20 nm/s; the data resolution was 1 nm/step; the initial wavelength was 200 nm, and final wavelength was 400 nm; the measurement type was remission and the measurement mode was absorption.

### 3.8. Validation of the NP-TLC Method

The proposed NP-TLC-densitometric method was validated by selectivity, linearity, accuracy, precision, limit of detection, limit of quantification and robustness according to the ICH guidelines [41], and according to the guidelines described by Ferenczi-Fodor et al. [46] and others guidelines [39,47,48,49,50].

#### 3.8.1. Selectivity and Resolution

The selectivity of the TLC method combined with densitometric analysis was based on the development of chromatographic separation of sodium diclofenac and its degradation products. The stability of examined compound was tested for acidic, alkaline, oxidation and photodegradation hydrolysis.

The peak resolution (*R_S(b)_*) was calculated using the equation [51,52]:

(1) $$R_{S(b)}=\frac{2d}{w_{b1}+w_{b2}},$$

where *d* is the distance between the centers of two adjacent peaks on the densitogram, whereas *w_b1_* and *w_b2_* are the peaks-width at the base.

##### Preparation of the standard solution of diclofenac sodium:

The standard solution for this study was a methanolic solution of diclofenac sodium made by dissolving 100 mg of diclofenac sodium in 25 mL of methanol. This resulted in primary solution of examined compound. From the solution obtained 1 mL was taken and supplemented with methanol to 10 mL. The solution obtained had a concentration of 4 mg/10 mL.

##### Acidic hydrolysis:

One milliliter was taken from the primary solution and next 1 mL of 1 M hydrochloric acid was added. The whole was made up to 10 mL with methanol (solution A).

##### Alkaline hydrolysis:

One milliliter was taken from the primary solution and next 1 mL of 1 M sodium hydroxide was added. The whole was made up to 10 mL with methanol (solution B).

##### Oxidation:

One milliliter was taken from the primary solution and next 0.5 mL of 30% hydrogen peroxide was added. The whole was made up to 10 mL with methanol (solution C).

##### Effects of UV radiation (254 nm):

One milliliter was taken from the primary solution and next the whole was made up to 10 mL with methanol (solution D).

Solutions A, B and C were heated at 90 °C for 2 h. Solution D was exposed to UV radiation at λ = 254 nm for 2 h. The solutions were then applied in the amount of 5 µL to plates coated with silica gel 60F_254_ (#1.05554).

To select the one that will enable the best separation of the above mentioned substances the following (11 mobile phases) were tested:

I: toluene:ethyl acetate:glacial acetic acid (60:40:1 *v*/*v*) [14],

II: toluene:ethyl acetate:methanol (4:4:2 *v*/*v*) [8],

III: toluene:acetone:glacial acetic acid (80:30:1 *v*/*v*) [13],

IV: toluene:acetone:glacial acetic acid (10:15:0,2 *v*/*v*) [5],

V: ethyl acetate:chloroform:methanol:ammonia (5:3.3:1.5:0,2 *v*/*v*) [6],

VI: dichloromethane:methanol:cyclohexane (95:5:40 *v*/*v*) [9],

VII: cyclohexane:chloroform:methanol (12:6:1 *v*/*v*) [7],

VIII: chloroform:methanol:ammonia (10:25:0,25 *v*/*v*) [10],

IX: toluene:acetonitrile:glacial acetic acid (60:50:2 *v*/*v*) [15],

X: hexane:chloroform:acetone:glacial acetic acid (60:60:30:1 *v*/*v*) [4],

XI: cyclohexane:chloroform:methanol:glacial acetic acid (6:3:0.5:0.5 *v*/*v*).

On this basis, after densitometric analysis, the three most effective mobile phases were selected for further studies of diclofenac sodium.

Selection of the optimal mobile phase from the mobile phases IV, X and XI to separate diclofenac from its acidic, alkaline, oxidation and UV degradation products

##### These studies were carried out as follows:

- Diclofenac sodium solution with added hydrochloric acid or sodium hydroxide and hydrogen peroxide, respectively was placed in a volume flask on hot plate at 90 °C. Every half hour the solution was cooled and then 5 μL of it was applied onto chromatographic plate. This action was repeated for 5 h. Chromatographic plates were then developed by using the three best mobile phases. The examined solution was then applied to the plates after 3 days, 10 days and 2, 3, 4 and 10 weeks. Each chromatographic plate was developed also by means of the three best mobile phases.
- In studies on the influence of UV radiation on the stability of diclofenac sodium, the methanolic solution of this compound was placed in a beaker under UV lamp (λ = 254 nm). Every half hour the solution was cooled and then 5 μL of it was applied onto chromatographic plate. This procedure was repeated for 5 h. Chromatographic plates were then developed by using the three best mobile phases. The examined solution was then applied to the plates after 3 days, 10 days and 2, 3, 4 and 10 weeks. Each chromatographic plate was developed by means of the three best mobile phases
- The stability of diclofenac sodium on silica gel was performed by applying the standard solution of this compound on two plates. One was placed under UV lamp, the other in a laboratory dryer at temperature of 40 °C. Each hour, a new portion of standard solution was spotted onto chromatographic plate, in such a way that the first portion was exposed to light and heated for 5 h. Each chromatographic plate was developed by means of the three best mobile phases

#### 3.8.2. Linearity and Range

The linearity of the TLC method was evaluated by analysis of standard solutions of diclofenac sodium at concentrations: 20.00; 18.00; 16.00; 14.00; 12.00; 10.00; 8.00; 6.00; 4.00; 2.00; 1.00 and 0.50 mg/5 mL. The solutions (5 μL) were applied to the same plate. The plates were developed using cyclohexane:chloroform:methanol:glacial acetic acid (6:3:0.5:0.5 *v*/*v*) mobile phase (XI) and scanned. The experiments were performed in six different analyses.

#### 3.8.3. Accuracy

This parameter was evaluated by measurement of recovery. A proper amount of diclofenac sodium standards in the low, medium and high regions of the calibration plots were added to powdered tablets of known active substance content. Next the tablets were extracted and analyzed under the optimized conditions. The experiments were performed in six different analyses.

#### 3.8.4. Precision

Intra-day precision of the method was verified by analysis of three replicates of three sample solutions (methanolic extracts of diclofenac sodium) at different concentrations under the same chromatographic conditions over a short interval of time (the same day). Inter-day precision was obtained for three sample solutions at different concentrations by an analyst who performed the analysis over a period of two weeks. To determine the precision of the procedure, the concentrations were prepared independently and experiments were performed in three different analyses. The precision of developed method was evaluated as the relative standard deviation (coefficient of variation, CV (%)).

#### 3.8.5. Limit of Detection and Limit of Quantification Based on the Calibration Curve

A specific calibration curve was studied using samples containing diclofenac sodium in the range of the limit of detection, namely 2.00, 1.00 and 0.50 μg/spot. This experiment was performed in six different analyses. The limit of detection (LOD) was calculated as:

(2) $$LOD=\frac{3.3\;\sigma }{S}.$$

The limit of quantification (LOQ) was calculated as:

(3) $$LOQ=\frac{10\;\sigma }{S},$$

where: σ—the standard deviation of the response, and S—the slope of the calibration curve.

#### 3.8.6. Robustness Study

Robustness was estimated by changing different chromatographic conditions in proposed procedure such as:

1. The kind of chromatographic plates (1.05554 and 1.05570);
2. Mobile phase volume (±5%);
3. Temperature of the activation of the plates at 120 (±5) °C;
4. Development distance (±5 mm);
5. Time of saturation of chromatographic chamber (±5 min).

The effect of these small, deliberate chromatographic conditions on the results (peak area of diclofenac sodium) was described.

### 3.9. Statistical Analysis

Statistical evaluation of the obtained results was performed with the use of the computer software Statistica 12.0.

## 4. Conclusions

The proposed new, simple NP-TLC method combined with densitometry is suitable for rapid and cost-effective qualitative as well as quantitative analysis of diclofenac sodium in commercial enteric tablets. The described method fulfilled all ICH guidelines requirements in term of validation. This method is selective, sensitive, accurate, precise and robust. The selectivity of the TLC method combined with densitometric analysis was used to develop a chromatographic separation of diclofenac sodium from its degradation products. Moreover, the stability of diclofenac sodium on acidic and alkaline hydrolysis, oxidation and photodegradation was tested. Silica gel 60F_254_ and cyclohexane:chloroform:methanol:glacial acetic acid (6:3:0.5:0.5 *v*/*v*) mobile phase allowed the separation of diclofenac and the largest number of diclofenac sodium degradation products (a total of 12 degradation products of diclofenac sodium). Linearity range was found to be 4.00–18.00 μg/spot for sodium diclofenac.

The results of the assay of enteric tablet formulations equaled 98.8% diclofenac sodium in relation to label claim was in good agreement with pharmacopoeial requirements.

It could be suggested that developed TLC-densitometric method may be used for the routine analysis of diclofenac sodium in pharmaceutical formulations. This method is suitable for analyzing of diclofenac sodium in tablet formulation without any interferences from additives present in pharmaceutical product. The developed method can be used to detect degradation products of diclofenac sodium in pharmaceutical preparations, because it enables the detection as recommended by the American Pharmacopoeia [36] diclofenac related compound A (1-(2,6-dichlorophenyl)-1,3-dihydro-2H-indol-2-one). Moreover, the proposed method allows us to detect more amount of impurities (compared to Starek and Krzek paper [7]).

The developed method may be an alternative to the diclofenac sodium studies described in the pharmacopoeias [36,37,38], especially if the laboratory does not have a highly efficient liquid chromatograph. Additionally, by comparing the R_F_ values and spectra of drug containing diclofenac sodium as well as its standard, the proposed method may be used to study its identity.

## Figures and Tables

**Table 1:** Method validation data for the quantitative determination of diclofenac sodium by Normal Phase Thin-Layer Chromatography (NP-TLC) with densitometry *^a^* after separation using cyclohexane: chloroform: methanol: glacial acetic acid (6:3:0.5:0.5 *v*/*v*).

	Method Characteristic	Results
	Selectivity	Selective
	Range (μg/spot)	4.00–18.00
	Linearity	A = 6918.2 (±147.6) + 274.6 (±12.4) × *n* = 8; r = 0.994; s = 160.6; F = 491.1; *p* < 0.0001
	Limit of detection (LOD; μg/spot) Limit of quantification (LOQ; μg/spot)	0.28 0.84
	For tablets Accuracy and precision	
	Accuracy (*n* = 6)	
	for 50% standard added (*n* = 6) for 100% standard added (*n* = 6) for 150% standard added (*n* = 6)	R = 100.4%; CV = 2.48% R = 100.0%; CV = 2.81% R = 99.4%; CV = 2.81%
	Quantity of Precision (CV, (%)) *n* = 3	
Interday	6.00 μg/spot	0.72
	10.00 μg/spot	1.54
	16.00 μg/spot	2.15
Intraday	6.00 μg/spot	1.26
	10.00 μg/spot	1.57
	16.00 μg/spot	2.69
Robustness	Robust	

*^a^* A = peak area (AU), x = amount (μg/spot) of drug analyzed, r = correlation coefficient, R = recovery (%), CV = coefficient of variation (%).

**Table 2:** Robustness of the proposed method (*n* = 5).

Parameter	% RSD of Peak Area
The kind of chromatographic plates	0.88
Mobile phase volume (±5%)	0.78
Temperature of the activation of the plates at 120 (±5) °C	0.82
Development distance (±5 mm)	1.35
Time of saturation of chromatographic chamber (±5 min)	0.89

**Table 3:** The statistical data concerning the results of the quantitative determination of diclofenac sodium in commercial enteric tablets examined by elaborated NP-TLC with the densitometry method.

Number of Analysis	Diclofenac Sodium Determined by NP-TLC with Densitometry
1	50.1
2	48.6
3	49.7
4	47.3
5	47.8
6	51.9
7	50.5
8	50.3
9	48.9
10	49.2
Average amount (mg/tablet)	49.4
The label claim (mg/tablet)	50
Standard deviation (SD)	1.36
Coefficient of variation (CV%)	2.75
Confidence interval of arithmetic mean with confidence level equal 95%	μ = 49.4 ± 1.0
Amount of diclofenac sodium (%) in relations to the label claim	98.8

## Supplementary Materials

The following are available online at https://www.mdpi.com/1424-8247/12/4/183/s1, Figure S1: Densitogram of tested solutions using mobile phase I. Figure S2: Densitogram of tested solutions using mobile phase II. Figure S3: Densitogram of tested solutions using mobile phase III. Figure S4: Densitogram of tested solutions using mobile phase IV. Figure S5: Densitogram of tested solutions using mobile phase V. Figure S6: Densitogram of tested solutions using mobile phase VI. Figure S7: Densitogram of tested solutions using mobile phase VII. Figure S8: Densitogram of tested solutions using mobile phase VIII. Figure S9: Densitogram of tested solutions using mobile phase IX. Figure S10: Densitogram of tested solutions using mobile phase X. Figure S11: Densitogram of tested solutions using mobile phase XI. Figure S12: Densitogram after separation using a mobile phase IV. Figure S13: Densitogram after separation using a mobile phase X. Figure S14: Densitogram after separation using a mobile phase IX. Figure S15: Calibration plot (A) and plot of residuals (B) for diclofenac sodium in the linear working range (mobile phase XI: cyclohexane: chloroform: methanol: glacial acetic acid, 6: 3: 0.5: 0.5 v/v). Table S1: RF and RS values of diclofenac sodium with the addition of hydrochloric acid, which was heated at 90 °C for 90 min and developed in the XI mobile phase. Table S2: RF and RS values of diclofenac sodium with the addition of hydrochloric acid, which was heated at 90 °C for 5 h and developed in the XI mobile phase. Table S3: RF and RS values of diclofenac sodium from its methanolic solution, which was exposed to UV radiation (λ = 254 nm) for 5 h and developed in the XI mobile phase. Table S4: RF and RS values of diclofenac sodium, which was exposed to UV radiation (λ = 254 nm) on silica gel for 5 h and developed in the XI mobile phase.
