# Supplementary material for: Application of Thin-Layer Chromatography in Combination with Densitometry for the Determination of Diclofenac in Enteric Coated Tablets
Source: Pharmaceuticals (Basel). 2019 Dec 16;12(4):183. doi: 10.3390/ph12040183 (PMC6958427; doi:10.3390/ph12040183)
Supplement: Supplementary file 1 [file pharmaceuticals-12-00183-s001.pdf]

Mobile phases used

To select the one that will enable the best separation of the above mentioned substances the following (11 mobile phases) were tested:

I: toluene: ethyl acetate: glacial acetic acid (60: 40: 1 v/v),
II: toluene: ethyl acetate: methanol (4: 4: 2 v/v),
III: toluene: acetone: glacial acetic acid (80: 30: 1 v/v),
IV: toluene: acetone: glacial acetic acid (10: 15: 0,2 v/v),
V: ethyl acetate: chloroform: methanol: ammonia (5: 3,3: 1,5: 0,2 v/v),
VI: dichloromethane: methanol: cyclohexane (95: 5: 40 v/v),
VII: cyclohexane: chloroform: methanol (12: 6: 1 v/v),
VIII: chloroform: methanol: ammonia (10: 25: 0,25 v/v),
IX: toluene: acetonitrile: glacial acetic acid (60: 50: 2 v/v),
X: hexane: chloroform: acetone: glacial acetic acid (60: 60: 30: 1 v/v),
XI: cyclohexane: chloroform: methanol: glacial acetic acid (6: 3: 0,5: 0,5 v/v).

To select mobile phases, test solutions were prepared as described in Methodology, solutions A, B, C were heated for 2 hours, solution D was exposed to UV radiation (λ=254 nm) for 2 hours. The solutions (5 μL) were spotted on the chromatographic plates.

Figures 1-11 show densitograms of the tested solutions of diclofenac sodium in various mobile phases: track 1- standard, track 2- with the addition of hydrochloric acid, track 3- with the addition of sodium hydroxide, track 4- with the addition of hydrogen peroxide, track 5- sample irradiated with radiation UV

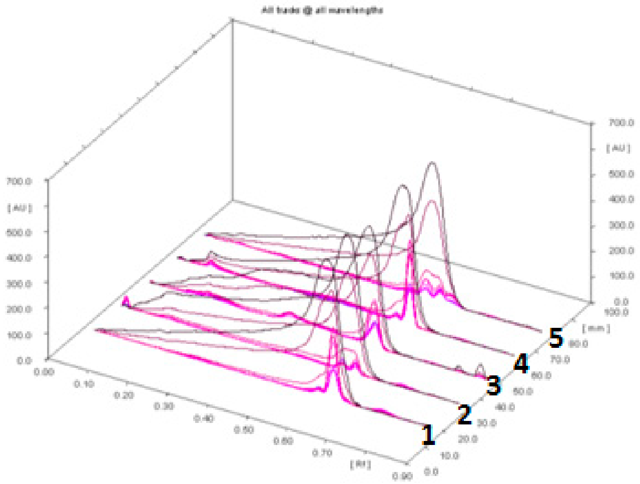

Fig. 1 Densitogram of tested solutions using mobile phase **I**.

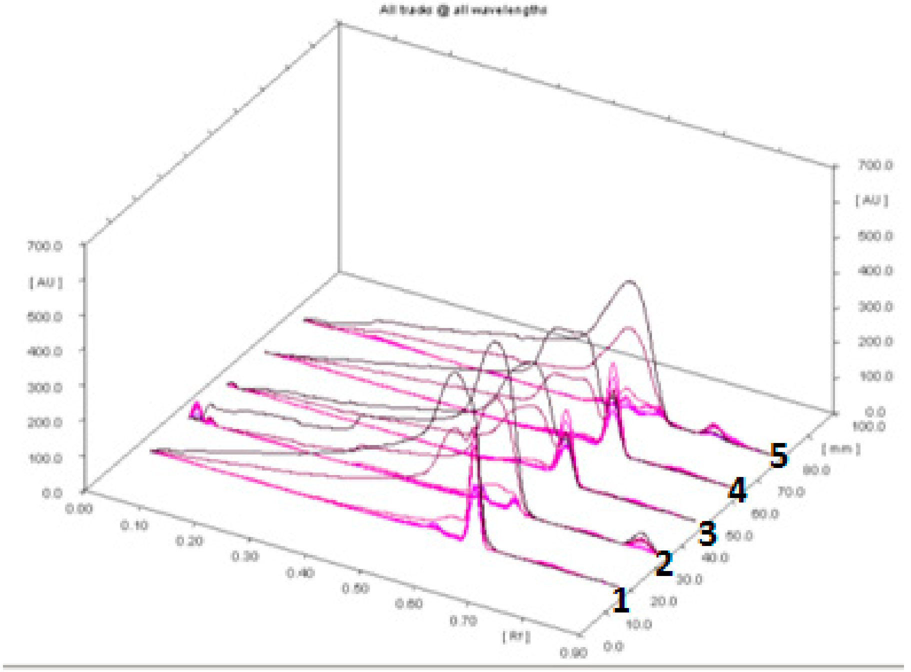

Fig. 2 Densitogram of tested solutions using mobile phase **II**

Fig. 3 Densitogram of tested solutions using mobile phase **III**

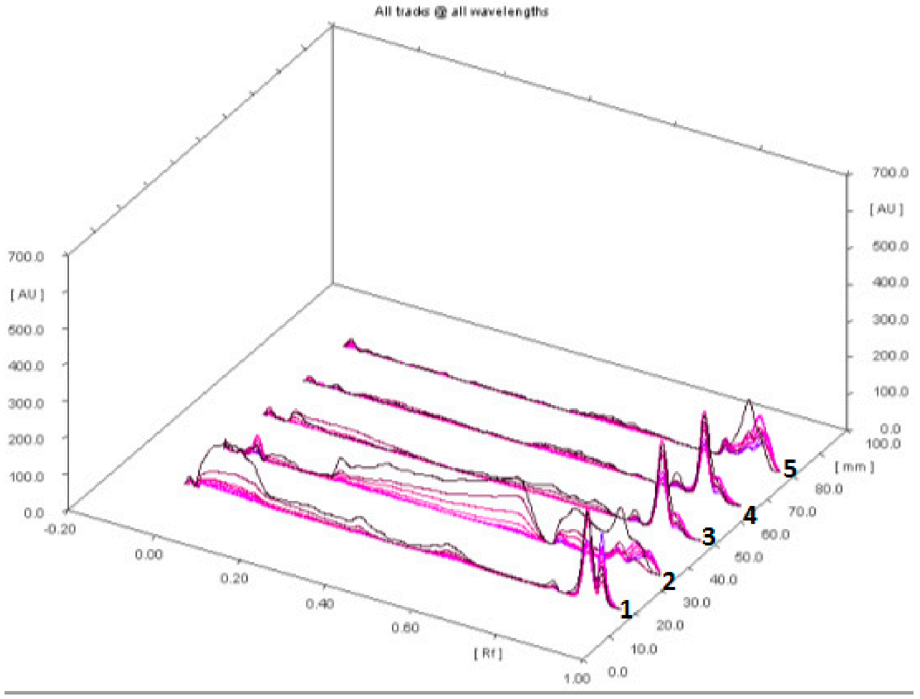

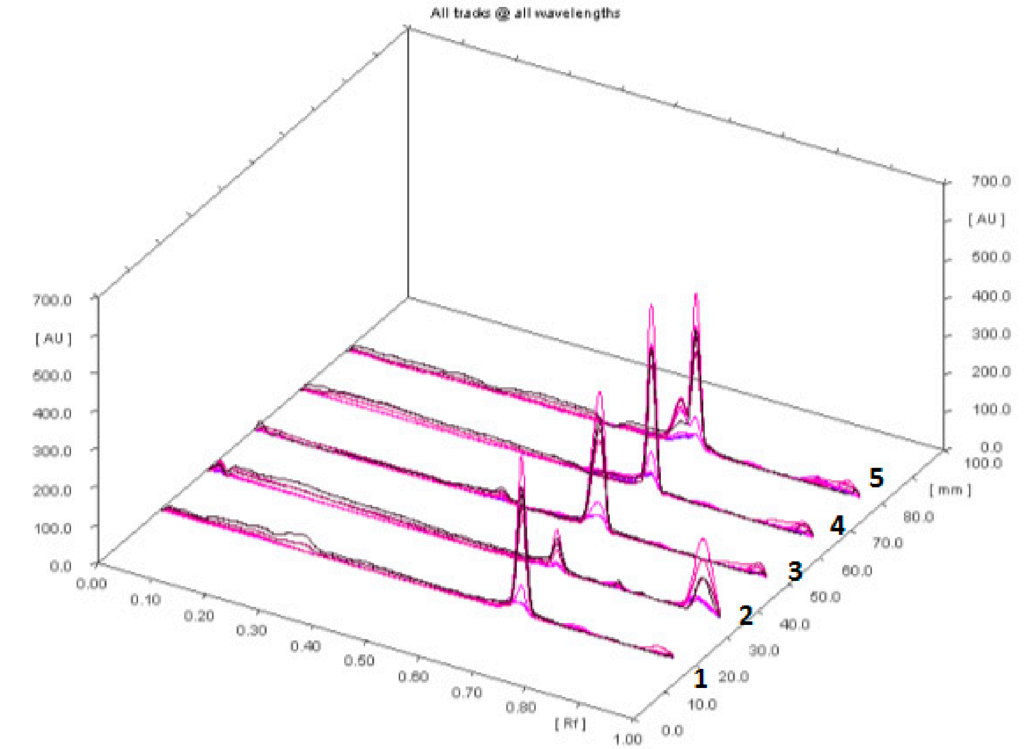

Fig. 4 Densitogram of tested solutions using mobile phase **IV**

Fig. 5 Densitogram of tested solutions using mobile phase **V**

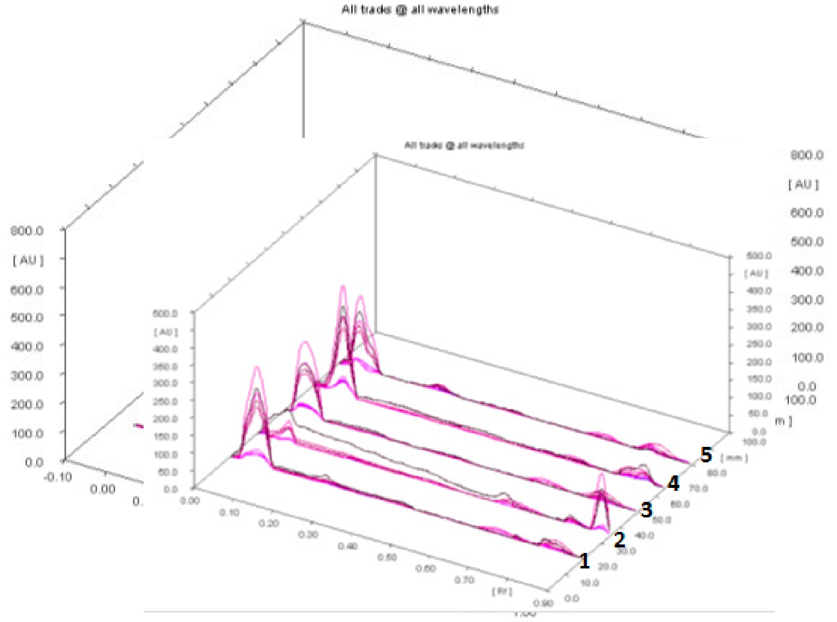

Fig. 6 Densitogram of tested solutions using mobile phase **VI**

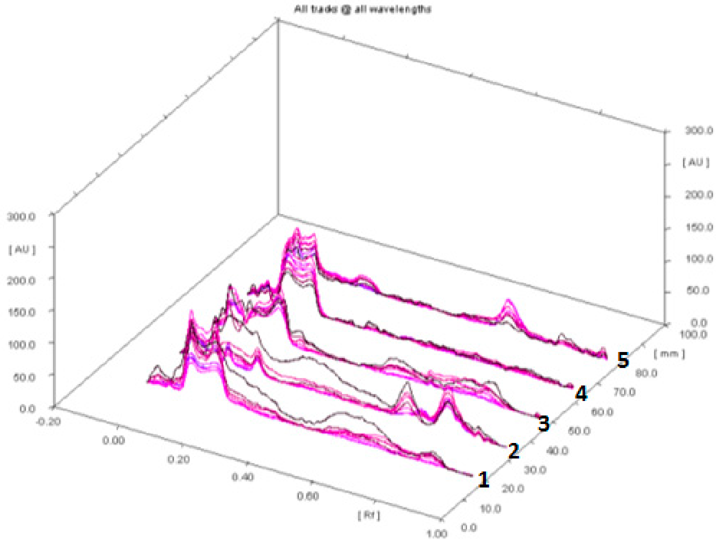

Fig. 7 Densitogram of tested solutions using mobile phase **VII**

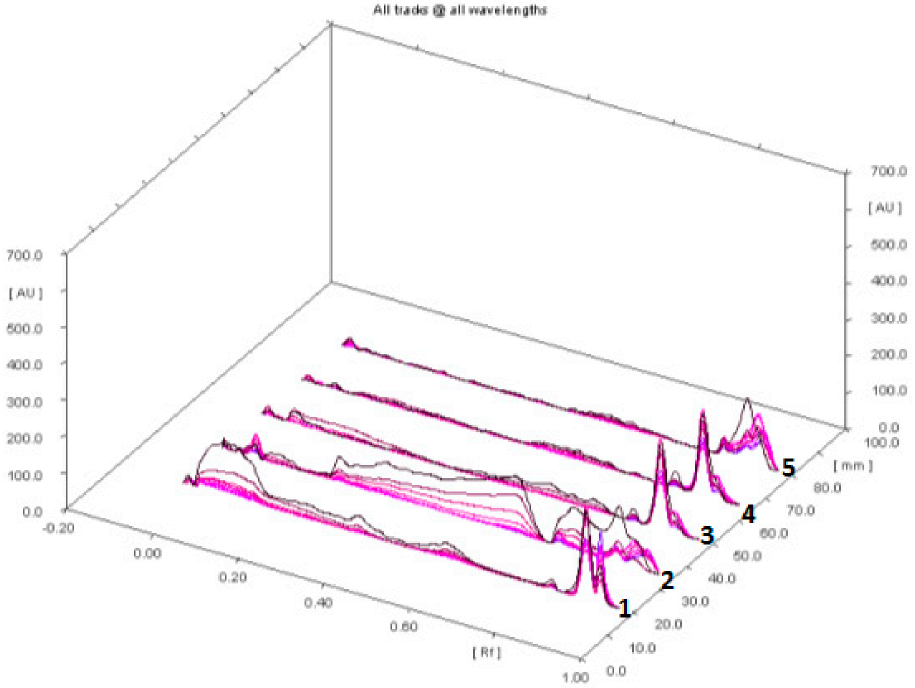

Fig. 8 Densitogram of tested solutions using mobile phase **VIII**

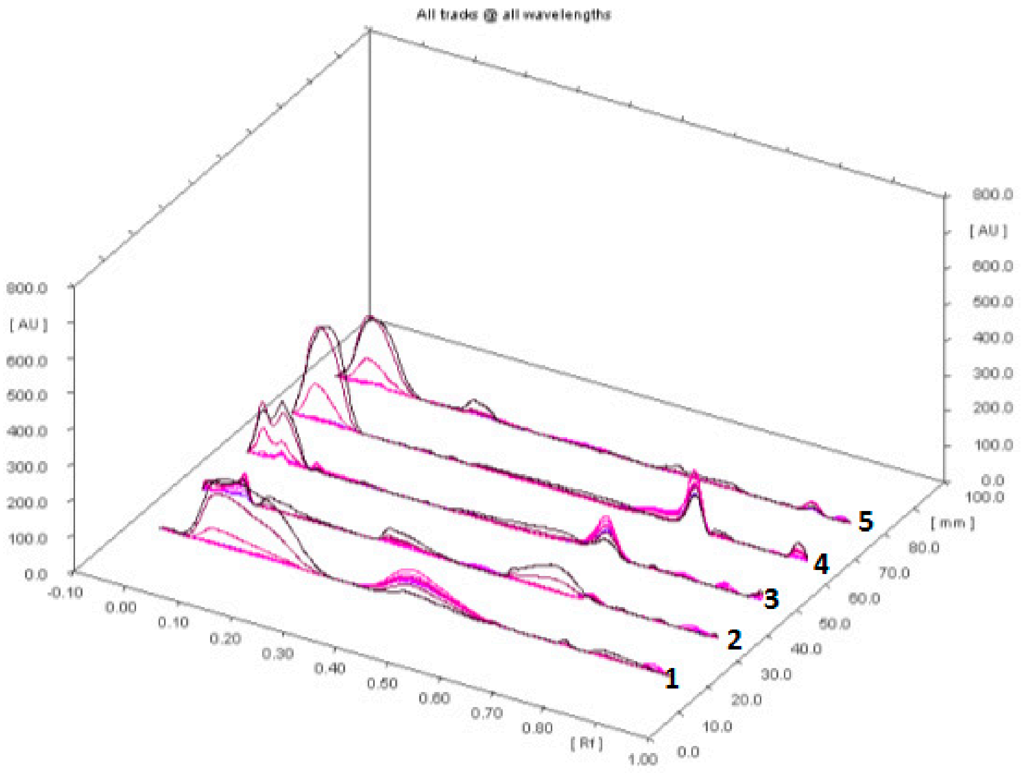

Fig. 9 Densitogram of tested solutions using mobile phase **IX**

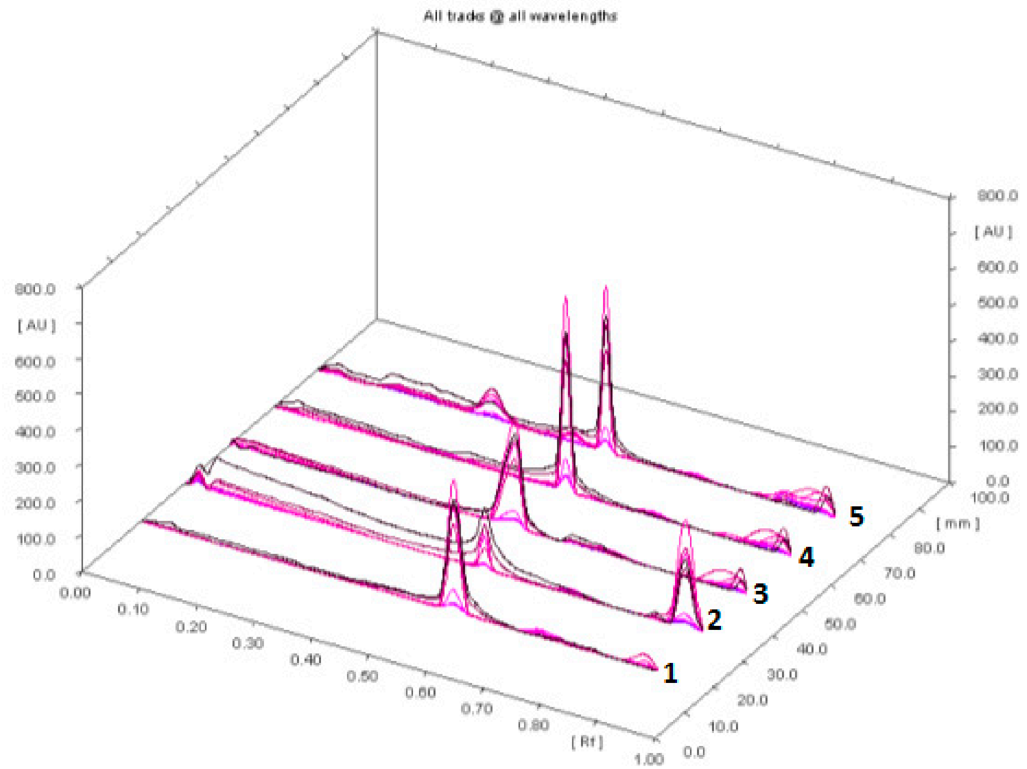

Fig. 10 Densitogram of tested solutions using mobile phase **X**

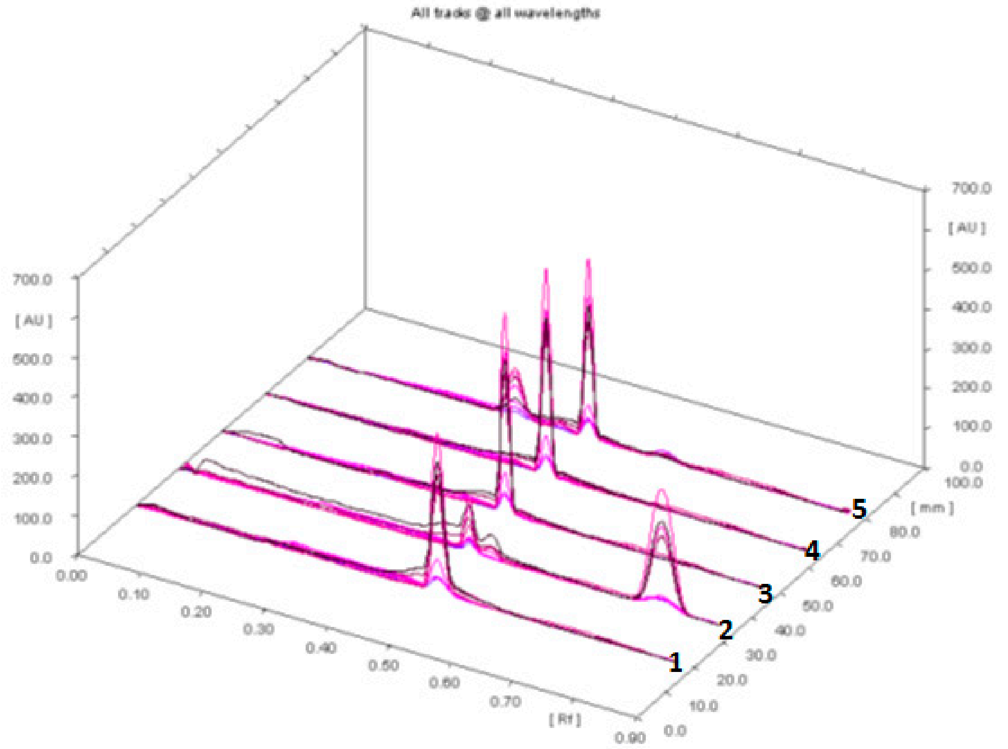

Fig. 11 Densitogram of tested solutions using mobile phase **XI**

Based on the above analysis results, 3 mobile phases were selected for further research, namely phases: IV, X, XI

Methanolic solution of diclofenac sodium with the addition of HCl, heated at 90 ° C

1- Immediately after adding HCl
2- 0.5 hours heated
3- 1.0 hours heated
4- 1.5 hours heated
5- 2.0 hours heated
6- 2.5 hours heated
7- 3.0 hours heated
8- 3.5 hours heated
9- 4.0 hours heated
10- 4.5 hours heated
11-5 hours heated
12- Standard, i.e. methanolic solution of diclofenac sodium

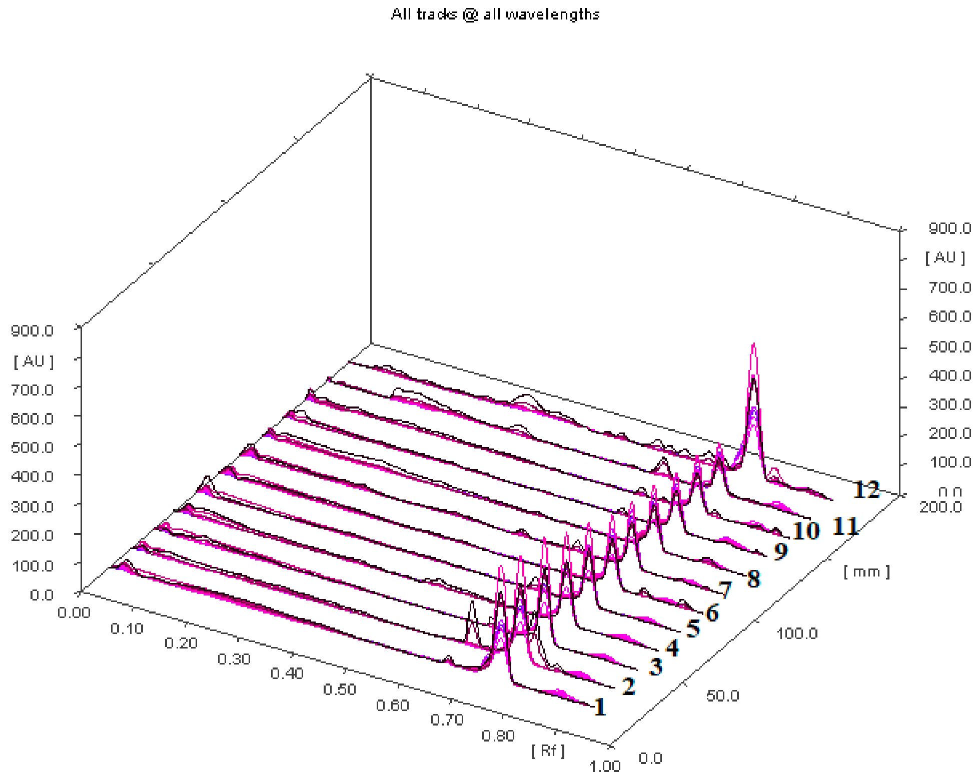

Fig. 12 Densitogram after separation using a mobile phase IV

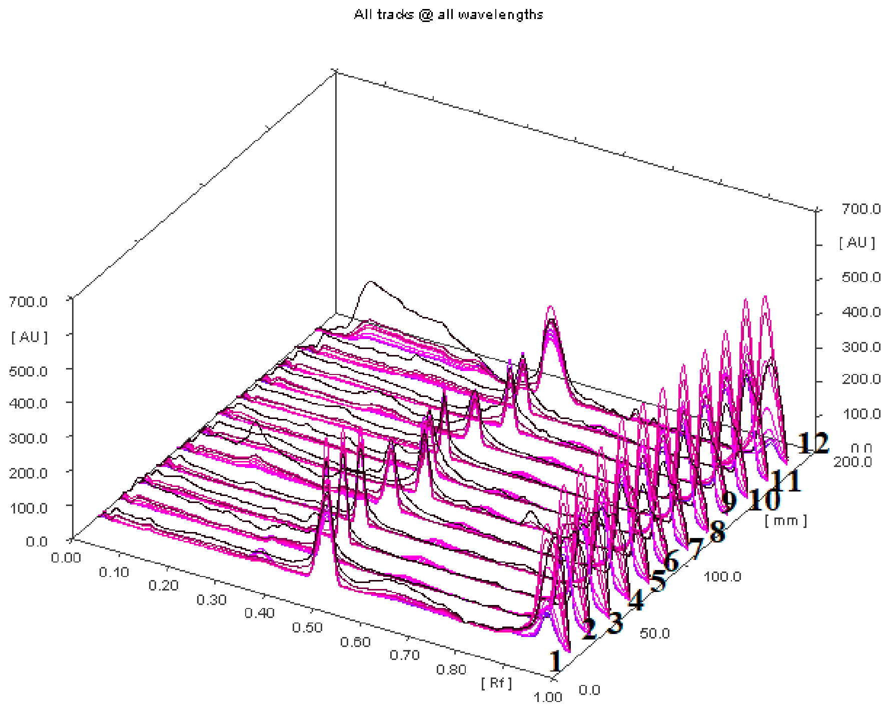

Fig. 13 Densitogram after separation using a mobile phase X

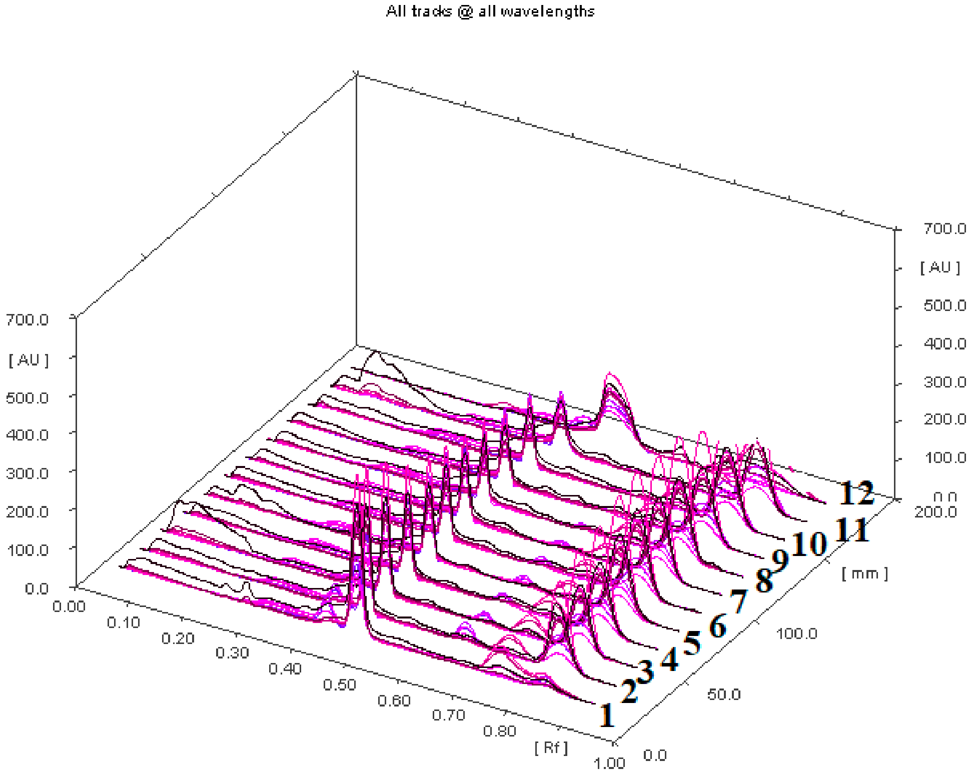

Fig. 14 Densitogram after separation using a mobile phase IX

CALIBRATION

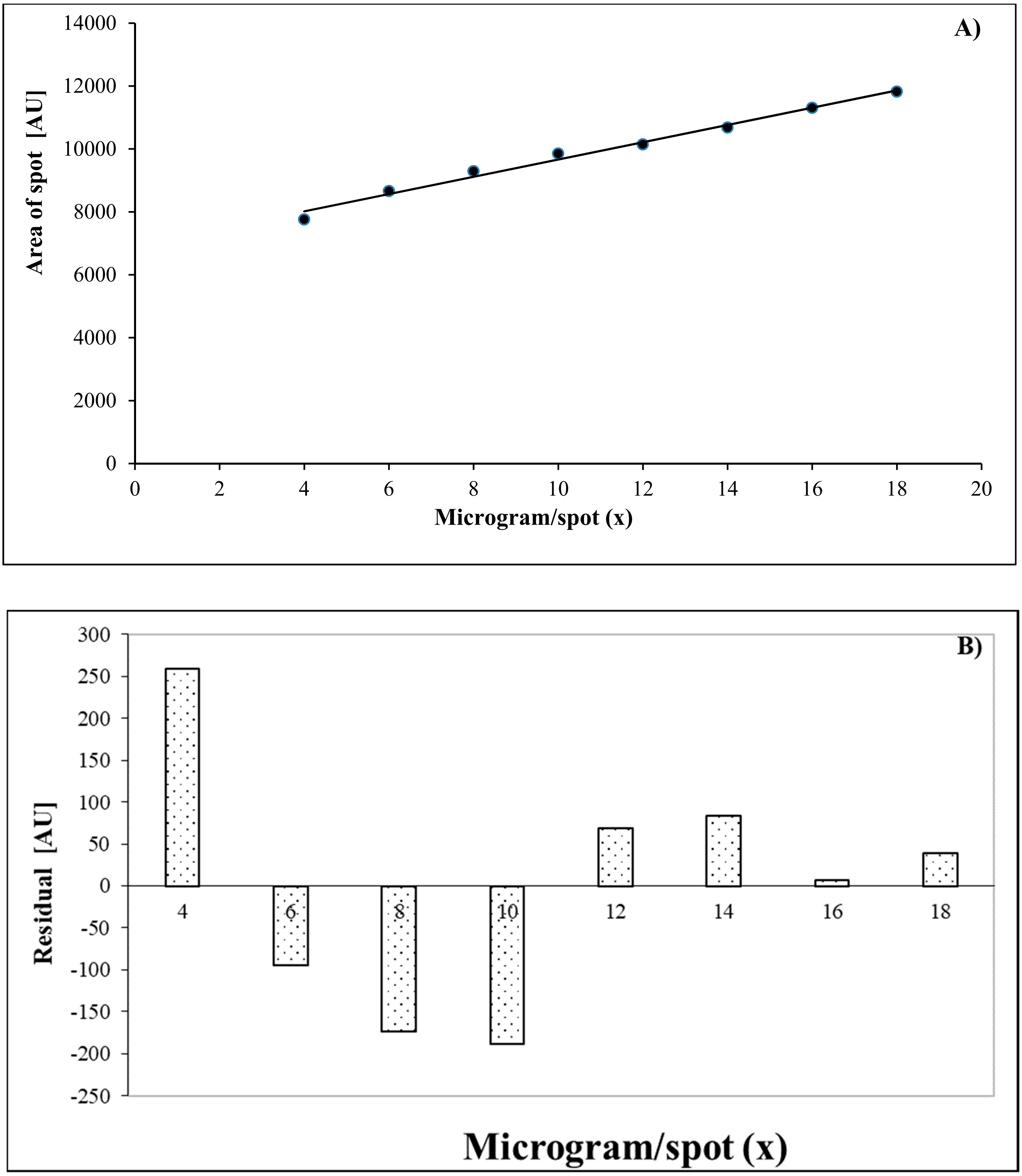

Fig. 15 Calibration plot (**A**) and plot of residuals (**B**) for diclofenac sodium in the linear working range (mobile phase **XI:** cyclohexane: chloroform: methanol: glacial acetic acid, 6: 3: 0,5: 0,5 v/v).

Table 1. R_F and R_S values of diclofenac sodium with the addition of hydrochloric acid, which was heated at 90ºC for 90 minutes and developed in the XI mobile phase (D- diclofenac sodium, A-diclofenac related compound A [1-(2,6-dichlorophenyl)-1,3-dihydro-2H-indol-2-one], P - unidentified degradation products of diclofenac sodium (see Fig.1 in the manuscript)

Symbol compound	R_F	R_S
P	0.11	
P	0.38	2.40
D	0.45	1.33
P	0.63	4.54
P	0.73	1.60
A	0.80	1.60

Table 2. R_F and R_S values of diclofenac sodium with the addition of hydrochloric acid, which was heated at 90°C for 5 h and developed in the XI mobile phase (D- diclofenac sodium, A-diclofenac related compound A [1-(2,6-dichlorophenyl)-1,3-dihydro-2H-indol-2-one], P - unidentified degradation products of diclofenac sodium (see Fig.2 in the manuscript)

Symbol compound	R_F	R_S
P	0.07	
P	0.11	1.05
P	0.16	0.33
D	0.45	4.16
P	0.73	4.40
A	0.80	0.89

Table 3. R_F and R_S values of diclofenac sodium from its methanolic solution, which was exposed to UV radiation (λ = 254 nm) for 5 h and developed in the XI mobile phase (D- diclofenac sodium, P - unidentified degradation products of diclofenac sodium (see Fig.3 in the manuscript)

Symbol compound	R_F	R_S
P	0.18	
P	0.37	1.83
P	0.42	1.23
D	0.45	1.56
P	0.65	3.76
P	0.91	4.20

Table 4. R_F and R_S values of diclofenac sodium, which was exposed to UV radiation (λ = 254 nm) on silica gel for 5 h and developed in the XI mobile phase (D- diclofenac sodium, P - unidentified degradation products of diclofenac sodium (see Fig.4 in the manuscript)

Symbol compound	R_F	R_S
P	0.03	
P	0.21	4.60
P	0.33	2.71
P	0.41	1.18
D	0.45	0.92
P	0.75	3.62
P	0.91	2.31
